# Cooccurrence of CD10-Positive and CD10-Negative Mantle Cell Lymphoma Complicated With Central Nervous System Involvement Solely by CD10-Positive Population

**DOI:** 10.7759/cureus.21341

**Published:** 2022-01-17

**Authors:** Noriyasu Fukushima, Tatsuji Mino, Koji Arihiro, Tatsuo Ichinohe

**Affiliations:** 1 Hematology and Oncology, Research Institute for Radiation Biology and Medicine, Hiroshima University, Hiroshima, JPN; 2 Hematology, Hiroshima University, Hiroshima, JPN; 3 Anatomical Pathology, Hiroshima University, Hiroshima, JPN

**Keywords:** bendamustine, cns relapse, discordant phenotype, cd10, mantle cell lymphoma

## Abstract

The neoplastic cells of mantle cell lymphoma (MCL) usually express CD5 and not CD10. However, cases of MCL with aberrant expression of CD10 have been seldom reported. A 71-year-old man presented multiple lymphadenopathies with a bulky tumor of the abdomen. He received the biopsies from the left cervical lymph node and the duodenum. The former specimen showed MCL with CD5-positive and CD10-negative, but the latter showed MCL with CD5-positive and CD10-positive. After receiving induction therapy, he developed convulsions, and lymphoma cells expressing CD5-positive and CD10-positive were detected in cerebrospinal fluid (CSF). CD10-positive MCL has some significant clinical characteristics. And it shows worse overall survival compared with CD10-negative MCL when it has aggressive features such as blastoid and pleomorphic morphology, high-Ki-67 index, and high mantle cell lymphoma international prognostic index (MIPI). Therefore, physicians and pathologists must carefully discriminate against cases having this aberrant expression.

## Introduction

Mantle cell lymphoma is an aggressive B-cell lymphoma that harbors t(11;14) (q13;q32), involving cyclin D1 and immunoglobulin (Ig) heavy chain genes. This genetic alteration is present in more than 95% of mantle cell lymphoma (MCL) cases and results in dysregulated overexpression of cyclin D1. The neoplastic cells of this lymphoma subtype usually express CD5 but not CD10. Cases of MCL with aberrant CD10 expression have rarely been reported [1]. Although largely unclear, MCL with aberrant CD10 expression is considered to have different biological and clinical characteristics than classical MCL. Herein, we present a case of co-occurrence of CD10-positive and CD10-negative MCL complicated with central nervous system (CNS) involvement solely by the CD10-positive population.

## Case presentation

**Figure 1:**
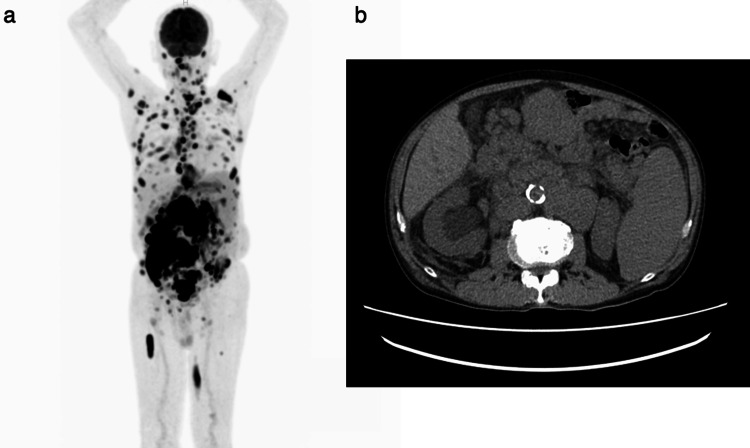
Positron emission tomography (a) and abdomen computed tomography (b). High 18F-fluorodeoxyglucose uptake in many swollen lymph nodes and bulky tumors of the abdomen was observed. Bulky abdominal tumors invaded the right ureter and induced hydronephrosis. Splenomegaly was also noted.

A 71-year-old man attended our hospital at the end of July 2020 to treat atrial fibrillation (AF). Ablation therapy was planned for the AF. However, he was diagnosed with multiple lymphadenopathy and anemia at that time, and ablation therapy was postponed. Positron emission tomography-computed tomography (PET-CT) revealed high 18F-fluorodeoxyglucose uptake in the swollen lymph nodes and a bulky tumor in the abdomen (Figure 1) complicated with right hydronephrosis.

**Figure 2:**
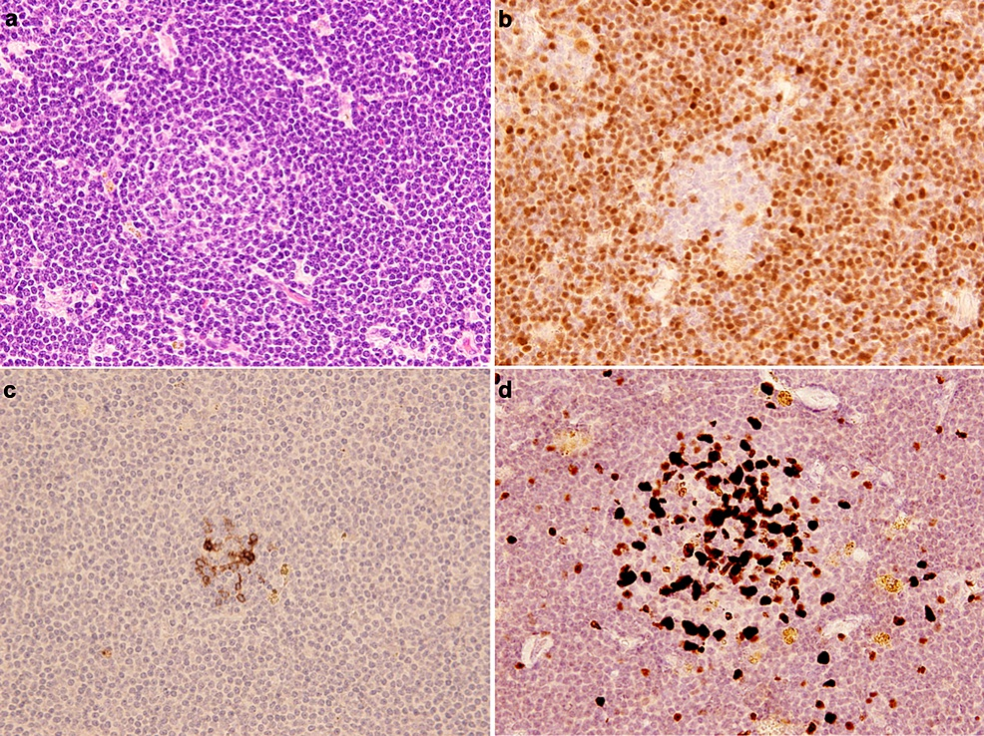
Left cervical lymph node biopsy specimen (a) Diffuse proliferation of medium cell-sized lymphoma cells was observed around the follicle (so-called naked follicle). The nuclei of these cells showed dispersed chromatin (Hematoxyline & Eosin stain). (b,c) Tumor cells around the follicle were positive for cyclinD1 (b) and negative for CD10 (c). (d) Ki-67 expressing cells were observed in the remnant follicle center, but most lymphoid cells around the follicle were not expressing.

Moreover, upper gastrointestinal endoscopy revealed slight erosion of the duodenum. We performed a biopsy of the left cervical node and a duodenal biopsy to obtain a definitive diagnosis. The cervical lymph node showed diffuse architectural effacement and some naked follicles (Figure 2a). Monotonous proliferation cells were medium-sized lymphoid cells whose nuclei showed dispersed chromatin. These cells expressed CD20, CD5, SOX-11, and cyclinD1 by immunohistochemistry but not CD10, CD23, and BCL-6. The Ki-67-positive tumor fraction was about 10% (Figure 2b-2d). G-band chromosomal analysis revealed t(11;14)(q13;q32) and trisomy 3 [17/20], and fluorescence in situ hybridization (FISH) revealed CCND1-IgH fusion signals. BCL-2-IgH fusion signal and TP53 alteration signal in FISH were not observed. We diagnosed classical mantle cell lymphoma (MCL) based on these findings.

**Figure 3:**
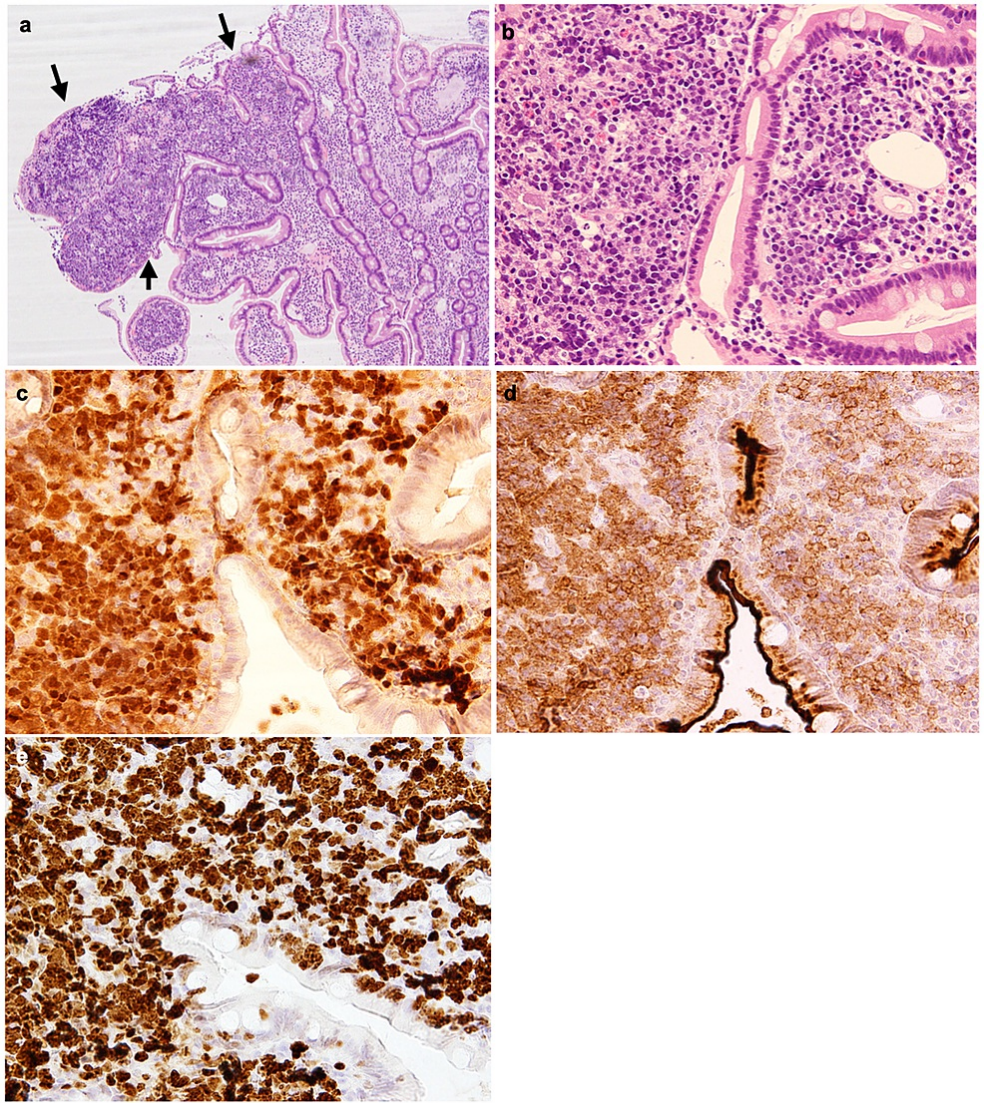
Duodenal biopsy specimen The dense proliferation of atypical lymphoid cells in part of the lamina propria was observed (arrows) (a, original magnification x40) The nuclei of lymphoma cells showed dense chromatin (b, original magnification x400). Lymphoma cells were positive for CD10 (c) and cyclin D1(d). The rate of Ki-67 expressing lymphoma cells is more than 70% (e).

On the other hand, the duodenal specimen showed compact proliferation of atypical medium to large-sized lymphoid cells in a part of the lamina propria (Figure 3a-3b). Blastic and pleomorphic morphology was inconspicuous. Intriguingly, these cells expressed CD10 and focally BCL-6, as well as CD20, CD5, SOX-11, and cyclin D1. The Ki-67-positivity was more than 70% (Figure 3c-3e). TP53 was negative in both specimens. We could not check other chromosomal and genetic examinations. Therefore, these pathological findings suggested the coexistence of MCL with different clinical and biological characteristics.

**Figure 4:**
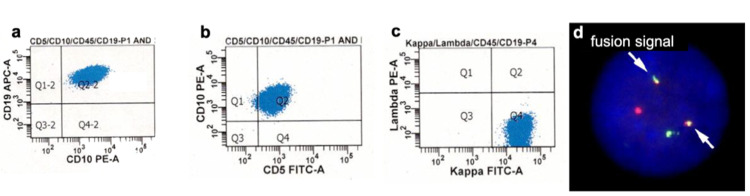
Flow cytometry and CCND1-IgH by FISH in the cerebrospinal fluid. Most lymphoma cells expressed CD19+/CD5+/CD10+ (a-c) and IgH-CCND1 fusion signals (arrows) (d). FISH: fluorescence in situ hybridization; IgH: immunoglobulin H

We initiated treatment with R-CHOP (rituximab, cyclophosphamide, doxorubicin hydrochloride (Hydroxydaunomycin), vincristine sulfate (Oncovin), prednisone), which led to gradual regression of the enlarged lymph nodes and the abdominal mass. Nevertheless, systemic convulsions occurred on day 17 of R-CHOP therapy. In addition, lumbar puncture revealed infiltration of lymphoma cells in the cerebrospinal fluid (CSF). These cells were positive for CD19, CD5, CD10, and surface immunoglobulin (Ig) kappa chain (Figure 4a-4c), with CCND1-IgH fusion (Figure 4d).

He received R-BAC500 (rituximab, bendamustine, and cytarabine [500 mg/m^2^]) [2] combined with intrathecal methotrexate injection. Complete metabolic remission was achieved, and the lymphoma cells disappeared in the CSF after six cycles of R-BAC500. However, he developed right facial nerve palsy one month after the end of R-BAC500. Lymphoma cells expressing CD5 and CD10 were present in the CSF, and central nervous system recurrence was confirmed. We started administration of ibrutinib (280 mg) as salvage therapy. However, he developed atrial fibrillation with tachycardia three days after administration and had multifocal cerebral infarction. He died nine months after the diagnosis.

## Discussion

Because of limited studies, the clinicopathological features of CD10-positive MCL are still unclear. Previous reports have suggested CD10-positive MCL is associated with blastoid and pleomorphic variants, high Ki-67 index, and high Mantle Cell Lymphoma International Prognostic Index (MIPI) [1,3]. CD10 expression in MCL with these aggressive features was significantly associated with worse overall survival [3]. In our patient, two discordant phenotypes that presented CD5-positive/CD10-negative and CD5-positive/CD10-positive were simultaneously observed, and the Ki-67 index of each lymphoma was distinctly different.

Immunophenotypic characteristics of CD10-positive MCL were not significant, except for one report [3]. This study described BCL-6 expression as higher in CD10-positive MCL (31%) than in CD10-negative MCL (7%). On the other hand, some studies that consisted of a small number of cases reported that BCL-6 positivity was about 11-20% in CD10-positive MCL [1,4].

It is essential to prove whether both lymphomas were the same clone or not. A few cases that acquired the CD10 antigen associated with histological transformation at relapse have been previously reported [5-7]. Since genetical clonality analysis at diagnosis and relapse was not performed in these cases, there was no evidence that lymphoma cells that acquired the CD10 antigen were the same clone at the time of diagnosis. Zanetto et al. observed sequence analysis of the immunoglobulin heavy chain variable region (IGVH) in five cases of CD10-positive MCL [5]. The evidence of immunoglobulin somatic hypermutation suggests that the neoplasm is a germinal center derivation. The result showed IGVH unmutated in four cases and lower revel mutation than typically seen in germinal center neoplasms in one case and suggested that CD10-positive MCL was not a derivation from a germinal center cell. On the other hand, Hadzidimitriou et al. reported that 13.8% of MCL had high somatic hypermutation [8]. Akhter et al. analyzed immunoglobulin somatic hypermutation status and gene expression profile data in CD10-positive MCL [4]. They showed that some cases of CD10-positive MCL had immunoglobulin somatic hypermutation and higher expression of germinal center-associated genes compared with CD10-negative MCL. They explained that CD10 expression in MCL might suggest a distinct ontology, not an aberrancy. Unfortunately, we could not gain the residual specimen of the duodenum to perform the additional study due to exhaustion of the diagnostic material. However, further investigation for MCL cases with discordant immunophenotype is necessary to understand the biological characteristics of CD10-positive MCL.

In this case, lymphoma cells infiltrating into the CNS were solely CD10-positive. Given that the Ki-67 index is a strong predictor of CNS relapse in MCL patients [9], high Ki-67 positivity of the CD10-positive population might be associated with a high risk of CNS involvement. Of note here, Morice et al. reported a case showing the coexistence of CD5-positive/CD10-negative classic MCL and CD5-negative/CD10-positive blastoid MCL, and the latter clones were only detected in CSF at relapse [6]. The evidence that only CD10-positive MCL cells were infiltrated to the CNS was similar to our case. On the other hand, Xu could not find a significant difference in the CNS involvement rate between CD10-positive and -negative MCL in a small number of cases [3]. Further investigation is necessary to clarify the relation between CD10-positive MCL and CNS infiltration.

Treatment of secondary CNS involvement of MCL is very challenging. Usually, a high-dose methotrexate-based regimen and/or intrathecal injection of methotrexate are used for these lymphomas because of well penetration into the CNS. Involvement of CNS in MCL is a rare complication [10], and treatment options are limited. A tissue distribution study in animals has shown that bendamustine was distributed broadly, including brain tissue with nearly two-thirds levels of non-brain organs [11-12]. Bendamustine with dexamethasone for a methotrexate-refractory recurrent primary CNS lymphoma showed complete and partial remission rates were 25% and 32%, respectively [12]. It is considered that R-BAC500 concurrent with intrathecal methotrexate had the optimal response for CNS. Ibrutinib is one of the promising options for MCL with CNS involvement. However, this agent sometimes induced atrial fibrillation, so we did not choose it first. Unfortunately, no durable response was observed, and atrial fibrillation deteriorated.

## Conclusions

The biological and clinicopathological features of CD10-positive MCL are still unclear; however, CD10-positive MCL may present a more aggressive clinical manifestation than the usual type of MCL. In addition, CD10-positive lymphoma cell clones may tend to infiltrate into the CNS compared with CD10-negative clones, according to the findings of our case. Therefore, physicians and pathologists must carefully discriminate against MCL patients having this aberrant expression.
